# Supplementary material for: Identification of Freezing-Responsive microRNAs and Their Targets in Chinese Jujube by Small RNA and Degradome Sequencing
Source: Int J Mol Sci. 2026 Sep 4;27(17):7912. doi: 10.3390/ijms27177912 (PMC13566890; doi:10.3390/ijms27177912)
Supplement: Supplementary file 1 [file ijms-27-07912-s001.zip › Supplementary Material S2/novel_miR_191.pdf]

Provisional ID	:	novel_miR_191
Score total	:	0.9
Score for star read(s)	:	-1.3
Score for read counts	:	0
Score for mfe	:	0.6
Score for randfold	:	1.6
Score for cons. seed	:	
Total read count	:	259
Mature read count	:	257
Loop read count	:	2
Star read count	:	0

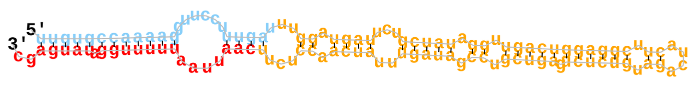

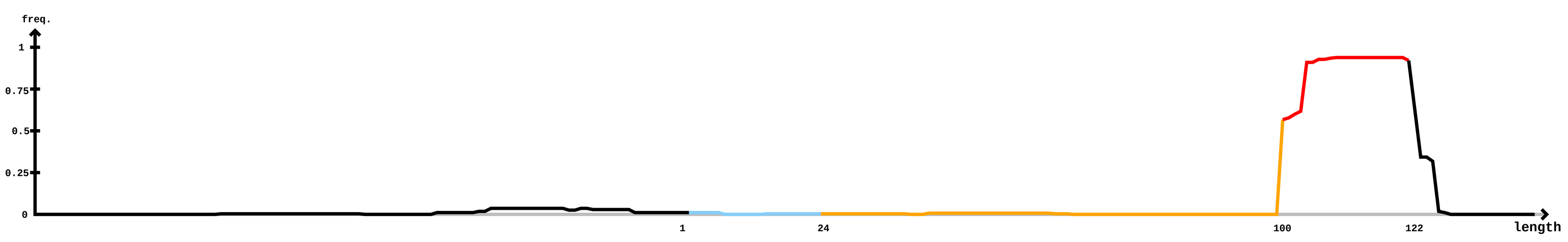

Star Mature

```
5'- ugaaauuuugaaaguaauuuuguacugucaaagauucuuucuguugauuaugauuuaguuagauccuaaaucauauggaucuugacuuucuugauagucggcccaucuugugccaaaagguuccuuugauuuggaugauucugcuauagguugacuggaggcuucaucagaugucucugagucguccgauaguuuaucaaccucuucaauuaauuuuuggauaugagcaagcaucuccuucuuugaucug -3'
.(((.((((((.((((.....)))).)))))).))).....((((((((.(...((((((((((.........)))))).)))))....).)))))))))....((((((((((((......((((...((.((((...(((((.((.((((((((((((.((....)).)))))).))))).)).))))).)))).))...))))....))))))).))))))..((.(((........))))).
```

read	reads	mm	sample
aaagauucCuucuguugauuauga	1	1	JCK-3
cuaaaucauaGggaucuugacu	3	1	LT-2
auauggaucuugacuuucu	2	0	LT-1
auggaucuugacuGucuugauagu	2	1	JCK-1
auggaucuugacuGucuugauagu	3	1	JCK-2
cuugauagucggcccaucuugugc	3	0	LT-2
uuccuuugauuuggaugauucugc	1	0	LT-1
agguugacuggaggcuucauc	1	0	LT-1
agguugacuggaggcuucaucaga	1	0	LT-3
caauuaauuuuuggauaugagc	9	0	LT-3
caauuaauuuuuggauaugagc	3	0	LCK-3
caauuaauuuuuggauaugagc	10	0	LT-2
caauuaauuuuuggauaugagc	16	0	LCK-2
caauuaauuuuuggauaugagc	17	0	JCK-1
caauuaauuuuuggauaugagc	2	0	LT-1
caauuaauuuuuggauaugagc	30	0	JCK-3
caauuaaCuuuuggauaugagc	1	1	LT-3
caauuaauuuuuggauaugagc	47	0	JCK-2
caauuaauuuuuggauaugagc	16	0	LCK-1
caauuaauuuuuggauaugagca	2	0	LCK-3
caauuaauuuuuggauaugagca	1	0	LT-1
caauuaauuuuuggauaugagca	3	0	JCK-2
aauuaauuuuuggauaugagc	2	0	JCK-1
aauuaauuuuuggauaugagc	1	0	JCK-3
auuaauuuuuggauaugag	4	0	JCK-2
auuaauuuuuggauaugag	1	0	JCK-1
auuaauuuuuggauaugagcaagc	1	0	LT-3
uuaauuuuuggauaugagcaag	1	0	JCK-1
uuaauuuuuggauaugagcaag	2	0	JCK-2
uuaauuuuuggauaugagcaag	2	0	JCK-3
uaauuuuuggauaugagcaag	1	0	JCK-2
uaauuuuuggauaugagcaag	1	0	JCK-3
uaauuuuuggauaugagcaagc	25	0	LCK-2
uaauuuuuggauaugagcaagc	8	0	JCK-3

```
                                                                                                                 Star                                                                                    Mature
ugaaauuuugaaaguaauuuuguacugucaaagauucuuucuguugauuaugauuuaguuagauccuaaaucauauggaucuugacuuucuugauagucggcccaucuugugccaaaagguuccuuugauuuggaugauucugcuauagguugacuggaggcuucaucagaugucucugagucguccgauaguuuaucaaccucuucaauuaauuuuuggauaugagcaagcaucuccuucuuugaucug
..................................................................................................................................................................................................................uaauuuuuggauaugagcaagc..................	2	0	LT-1
..................................................................................................................................................................................................................uaauuuuuggauaugagcaagc..................	1	0	LT-3
..................................................................................................................................................................................................................uaauuuuuggauaugagcaagc..................	18	0	LCK-1
..................................................................................................................................................................................................................uaauuuuuggauaugagcaagc..................	11	0	JCK-1
..................................................................................................................................................................................................................uaauuuuuggauaugagcaagc..................	14	0	JCK-2
....................................................................................................................................................................................................................auuuuuggauaugagcaagc..................	1	0	JCK-1
....................................................................................................................................................................................................................auuuuuggauaugagcaagc..................	2	0	LT-3
....................................................................................................................................................................................................................auuuuuggauaugagcaagcau................	1	0	JCK-1
....................................................................................................................................................................................................................auuuuuggauaugagcaagcau................	1	0	JCK-3
......................................................................................................................................................................................................................uuuuggauaugagcaagca.................	2	0	LT-3
.......................................................................................................................................................................................................................uuuggauaugagcaagcau................	1	0	LT-3
```
